# FTD/ALS Type 7-Associated Thr104Asn Mutation of CHMP2B Blunts Neuronal Process Elongation, and Is Recovered by Knockdown of Arf4, the Golgi Stress Regulator

**DOI:** 10.3390/neurolint15030063

**Published:** 2023-08-11

**Authors:** Remina Shirai, Mizuka Cho, Mikinori Isogai, Shoya Fukatsu, Miyu Okabe, Maho Okawa, Yuki Miyamoto, Tomohiro Torii, Junji Yamauchi

**Affiliations:** 1 Laboratory of Molecular Neurology, Tokyo University of Pharmacy and Life Sciences, Tokyo 192-0392, Japan; rshirai@toyaku.ac.jp (R.S.); miyamoto-y@ncchd.go.jp (Y.M.); 2 Department of Pharmacology, National Research Institute for Child Health and Development, Tokyo 157-8535, Japan; 3 Laboratory of Ion Channel Pathophysiology, Doshisha University Graduate School of Brain Science, Kyoto 610-0394, Japan; ttorii@mail.doshisha.ac.jp; 4 Diabetic Neuropathy Project, Tokyo Metropolitan Institute of Medical Science, Tokyo 156-8506, Japan

**Keywords:** CHMP2B, N1E-115 cell, neuronal differentiation, Arf4, Golgi stress

## Abstract

Frontotemporal dementia and/or amyotrophic lateral sclerosis type 7 (FTD/ALS7) is an autosomal dominant neurodegenerative disorder characterized by the onset of FTD and/or ALS, mainly in adulthood. Patients with some types of mutations, including the Thr104Asn (T104N) mutation of charged multivesicular body protein 2B (CHMP2B), have predominantly ALS phenotypes, whereas patients with other mutations have predominantly FTD phenotypes. A few mutations result in patients having both phenotypes approximately equally; however, the reason why phenotypes differ depending on the position of the mutation is unknown. CHMP2B comprises one part of the endosomal sorting complexes required for transport (ESCRT), specifically ESCRT-III, in the cytoplasm. We describe here, for the first time, that CHMP2B with the T104N mutation inhibits neuronal process elongation in the N1E-115 cell line, a model line undergoing neuronal differentiation. This inhibitory phenotype was accompanied by changes in marker protein expression. Of note, CHMP2B with the T104N mutation, but not the wild-type form, was preferentially accumulated in the Golgi body. Of the four major Golgi stress signaling pathways currently known, the pathway through Arf4, the small GTPase, was specifically upregulated in cells expressing CHMP2B with the T104N mutation. Conversely, knockdown of Arf4 with the cognate small interfering (si)RNA recovered the neuronal process elongation inhibited by the T104N mutation. These results suggest that the T104N mutation of CHMP2B inhibits morphological differentiation by triggering Golgi stress signaling, revealing a possible therapeutic molecular target for recovering potential molecular and cellular phenotypes underlying FTD/ALS7.

## 1. Introduction

During central nervous system development, neurons undergo continuous and dynamic morphogenesis [1,2,3,4,5,6], which involves neurite outgrowth and outgrowth, navigation of neuronal processes, and synaptogenesis to form neural networks [1,2,3,4,5,6]. Neurite and/or small process outgrowth is the first and essential step in establishing neural networks. However, the overall molecular mechanisms underlying various neuronal cell morphological differentiation stages, such as neurite outgrowth and elongation, are still not fully understood [1,2,3,4,5,6]. On the other hand, in neurological diseases, neuronal morphogenesis can be affected at various stages [1,2,3,4,5,6].

Frontotemporal dementia (FTD) and amyotrophic lateral sclerosis (ALS) are neurodegenerative diseases that overlap in clinical, genetic, and pathological presentation [7,8]. FTD is a common cause of early-onset dementia in people under 60 years, and it refers to a heterogeneous group of disorders that involve degeneration of the frontotemporal lobe. ALS is the most common neurodegenerative disorder and is characterized by the progressive atrophy and degeneration of upper and lower motor neurons. Therefore, FTD and ALS are now considered single-spectrum disorders. It is thought that one of the common molecular and cellular FTD/ALS pathological mechanisms is impaired proteostasis, especially for abnormal and/or inhibitory trafficking coupling endosome to lysosome [7,8]. One of the protein components that constitutes these proteostasis pathways is believed to be charged multivesicular body protein 2B (CHMP2B) [7,8,9,10]. CHMP2B is a core component of the endosomal sorting complex required for transport (ESCRT) machinery, and it coordinates the scission of intracellular membranes [7,8,9,10]. The ESCRT machinery recognizes ubiquitinated proteins at the membrane surface of endosomes or multivesicular bodies (MVBs) to couple endosomes and MVBs to lysosomes [7,8,9,10]. Invasion and budding of the intracellular limiting membrane result in the formation of MVB endocytic vesicles, into which ubiquitinated proteins are incorporated and either degraded via lysosomes or sorted back to the plasma membrane. “Exosomes” describe extracellular endoplasmic reticulum secreted via MVBs, whereas “microvesicles,” which are different from exosomes, describe endoplasmic reticulum budding directly from the plasma membrane. As expected, dysfunction of CHMP2B (e.g., due to various amino acid mutations) can result in moderate to severe neurological diseases, including the FTD/ALS spectrum [7,8,9,10].

FTD/ALS type 7 is an autosomal dominant neurodegenerative disorder characterized by the onset of FTD and/or ALS primarily in adulthood, and it is caused by various positions of mutations in CHMP2B [7,8,11]. Patients with amino acid mutations including the Thr104Asn (T104N) mutation of CHMP2B have predominantly ALS phenotypes whereas patients with other mutations have predominantly FTD phenotypes [7,8,11]. A few mutations result in patients with both phenotypes [7,8,11]; however, the reason why phenotypes differ depending on the position of the mutation is unknown. Despite the relationship between mutations in CHMP2B and diseases, it is unclear whether and how a mutation in CHMP2B affects neuronal cells. In the present study, we describe, for the first time, that CHMP2B with the T104N mutation [12] greatly inhibits process elongation in the N1E-115 cell line, a widely used model of neuronal differentiation [13,14]. Cells expressing the T104N mutant protein caused Golgi stress, whereas a decrease in Golgi stress recovered the ability of cells to elongate processes, providing evidence of a potential pathological molecular and cellular mechanism underlying FTD/ALS7.

## 2. Materials and Methods

### 2.1. Key Antibodies and siRNA Sequences

The antibodies used in this study and the generated DNA plasmids are shown in Table 1. Sequences of 19-mer small interfering (si)RNAs using dTdT (Fasmac, Kanagawa, Japan) are shown in Supplemental Figure S2. Sequences of DNA primers (Fasmac) are shown in Supplemental Figure S2.

### 2.2. Reverse Transcription-Linked Polymerase Chain Reactions (RT-PCR)

cDNAs were prepared from total RNA extracted using Isogen solution (Nippon Gene, Tokyo, Japan) using the PrimeScript RT Master Mix and Perfect Real Time kit (Takara Bio, Kyoto, Japan) according to the manufacturers’ instructions.

PCR amplifications from reverse-transcribed single-strand polynucleotides were performed using Gflex DNA polymerase (Takara Bio) in 30 to 35 cycles consisting of denaturation reaction at 98 °C (0.2 min), an annealing reaction at 56–65 °C, depending on the respective annealing temperatures (0.25 min), and extension reaction at 68 °C (0.5 min). The resulting PCR products were applied to a 1–2% agarose gel (Nacalai Tesque, Kyoto, Japan).

### 2.3. Cell Line and Stable Clone Cultures and Differentiation

Mouse neuronal N1E-115 and green monkey kidney epithelial COS-7 cells (JCRB Cell Bank, Osaka, Japan, and Japan Health Sciences Foundation, Osaka, Japan) were cultured on 6- or 10-cm cell culture dishes (ThermoFisher Scientific, Waltham, MA, USA) in high-glucose Dulbecco’s modified Eagle medium (DMEM; Nacalai Tesque) containing 10% heat-inactivated fetal bovine serum (FBS; ThermoFisher Scientific) and penicillin-streptomycin solution (Nacalai Tesque) in 5% CO_2_ at 37 °C.

COS-7 and N1E-115 cells are high transfection efficiency and neuronal differentiation abilities, respectively [13,14]. Cell lines stably expressing the wild-type (indicated as WT in the figures) *Chmp2b* gene or the gene with the T104N mutation were selected in the presence of G418 (Nacalai Tesque) as described previously [15] and isolated as a single clone. To induce differentiation, N1E-115 cells were cultured in DMEM and 1% FBS containing penicillin-streptomycin in 5% CO_2_ at 37 °C for 48 h. Cells with processes longer than their cell body length were considered to be neurite-bearing cells (i.e., differentiated cells) [16]. Under these conditions, in each experiment, it was estimated that less than 5% of adherent cells incorporated trypan blue (Nacalai Tesque).

### 2.4. siRNA and Plasmid Transfection

COS-7 and N1E-115 cells were transfected with the generated plasmids and synthesized 21-mer siRNAs with dTdT using the ScreenFect A and ScreenFect siRNA transfection kit (Fujifilm, Tokyo, Japan) according to the manufacturer’s instructions, respectively. The medium was changed 4 h post-transfection and was typically used for 48 h post-transfection for cell biological and biochemical experiments. Under these conditions, it was estimated that less than 5% of adherent cells incorporated trypan blue.

### 2.5. Polyacrylamide Electrophoresis and Immunoblotting Techniques

Cells were lysed in lysis buffer [13,14,15,16]. Under normal denaturing conditions, cell lysates were denatured with sample buffer (Fujifilm), and samples were separated on sodium dodecyl sulfate-polyacrylamide gels (Nacalai Tesque). Separated proteins were transferred to polyvinylidene fluoride membranes (Fujifilm), blocked with Blocking One solution (Nacalai Tesque), and immunoblotted using a primary antibody followed by a peroxidase enzyme-conjugated secondary antibody. Peroxidase-reactive bands were captured using a CanoScan image scanner (Canon, Tokyo, Japan) and scanned using CanoScan software (Canon). The blots shown in the figure are representative of three blots. Several sets of experiments were performed in immunoblot studies, and Image J software (https://imagej.nih.gov/, accessed on 1 May 2023) was used to quantify immunoreactive bands with one control immunoreactive band as 100%.

### 2.6. Capturing Fluorescence Images

**Figure 1:**
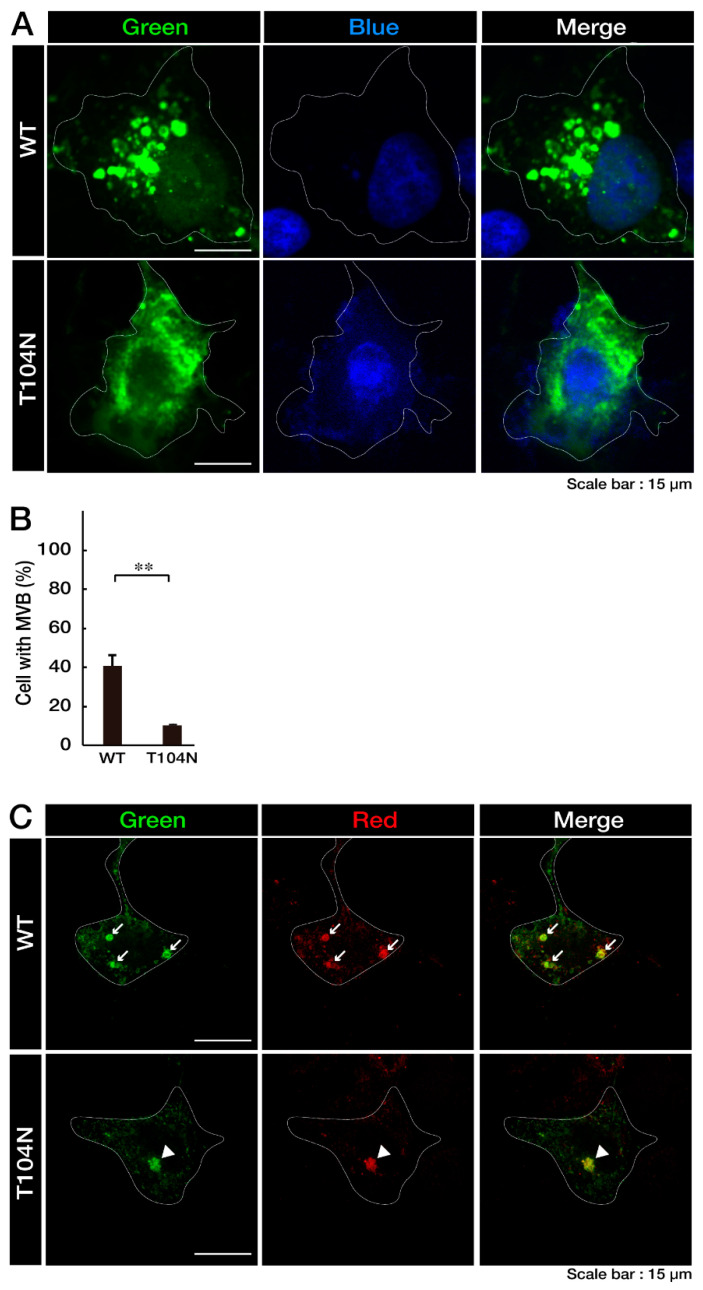
Wild-type CHMP2B is present in circular MVB-like structures in cells, whereas CHMP2B with the T104N mutation forms aggregate-like structures. (**A**,**B**) COS-7 cells (indicated by white dotted lines) were transfected with the plasmid encoding wild-type (WT) CHMP2B tagged with EGFP at its N-terminus or EGFP-tagged CHMP2B with the T104N mutation. Transfected cells (green) were stained with DAPI to detect nuclear positions (blue). Cells with circular MVB-like structures are statistically depicted in the graph (** *p* < 0.01; *n* = 10 fields). (**C**) COS-7 cells (indicated by white dotted lines) were transfected with the plasmid encoding wild-type (WT) CHMP2B tagged with EGFP at its N-terminus or EGFP-tagged CHMP2B with the T104N mutation. Transfected COS-7 cells were stained with ubiquitin (red). In the WT image, an MVB-like structure (indicated by the arrows) was observed. In the T104N mutation image, aggregate protein (indicated by the arrowhead) could be observed. MVB, multivesicular bodies; WT, wild-type; EGFP, enhanced green fluorescent protein; DAPI, 4′,6-diamidino-2-phenylindole.

Cells on coverslips were fixed with 4% paraformaldehyde (Nacalai Tesque) or 100% cold methanol (Nacalai Tesque) and blocked with Blocking One. Slides were incubated with a primary antibody followed by an Alexa-Fluor fluorescence-conjugated secondary antibody. Coverslips were mounted using the Vectashield kit (Vector Laboratories, Burlingame, CA, USA). The fluorescent images were collected and integrated on a microscope FV1200 or FV3000 system (both Olympus, Tokyo, Japan) equipped with a laser scanning Fluoview instrument and its software (Olympus). The image in Figure 1A is representative of three images and was analyzed using Image J software (https://imagej.nih.gov/, accessed on 1 May 2023).

### 2.7. Statistical Analyses

Values are shown as means ± standard deviation (SD) of separate experiments. Intergroup comparisons were made using an unpaired Student’s *t*-test in Excel (Microsoft, Redmond, WA, USA). A one-way analysis of variance (ANOVA) was followed by a Tukey’s multiple comparison test (MCT) using Graph Pad Prism (GraphPad Software ver. 5.0, San Diego, CA, USA). Differences were considered statistically significant when *p* < 0.05.

### 2.8. Ethical Approval

Techniques using genetically modified cells and related techniques were performed according to protocols approved by the Tokyo University of Pharmacy and Life Sciences Genetic Animal Control Committee (Approval Nos. LS28-20 and LSR3-011, verified on 1 April 2023).

## 3. Results

### 3.1. Wild-Type CHMP2B Is Contained in MVB-Like Structures Whereas T104N-Mutant CHMP2B Forms Aggregate-Like Structures

First, we confirmed that the wild-type (WT) CHMP2B protein is contained in MVB-like circular structures in COS-7 cells. Because COS-7 cells have a wide cytoplasmic region and are suitable for observing protein localization, we transfected plasmids carrying the WT CHMP2B or CHMP2B with the T104N mutation into COS-7 cells. Visual inspection showed that the WT CHMP2B protein was observed in the MVB-like structure (Figure 1A), whereas CHMP2B protein with the T104N mutation was primarily distributed in aggregate-like structures in the cytoplasm (Figure 1B), suggesting that the T104N mutation causes the CHMP2B protein form aggregates. Ubiquitin staining was performed to determine whether MVB or aggregation occurred. The ubiquitin was colocalized in the WT and T104N CHMP2B protein (Figure 1C). 

**Figure 2:**
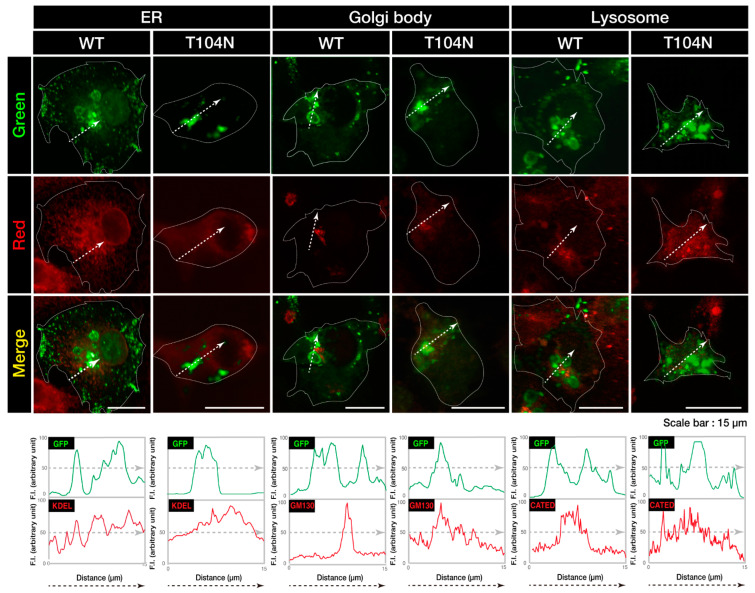
CHMP2B with the T104N mutation forms aggregate-like structures in the Golgi body. COS-7 cells were transfected with the plasmid encoding GFP-tagged wild-type (WT) or mutated CHMP2B (T104N). Transfected cells (green) were stained with an antibody against the ER-specific antigen KDEL (red), the Golgi body-specific antigen GM130 (red), or the lysosome-specific antigen cathepsin D (CATED, red). The approximate outlines of the cells are shown by white dotted lines. Scan plots were performed along the white lines in the direction of the arrows in the green and red images. Graphs showing fluorescence intensity (F.I.; arbitrary units) along the lines in the direction of the arrows are depicted in the bottom panels. GFP—green fluorescent protein; WT—wild-type; ER, endoplasmic reticulum.

Next, to determine which organelles were colocalized with aggregate-like structures of CHMP2B with the T104N mutation, we stained cells with different organelle markers. Mutated CHMP2B was colocalized with 130 kDa Golgi membrane protein (GM130) as the Golgi body marker, whereas WT CHMP2B was not (Figure 2). The endoplasmic reticulum (ER) marker Lys-Asp-Glu-Leu (KDEL) and the lysosome-specific antigen cathepsin D were not significantly colocalized with either WT CHMP2B or with the mutant’s aggregate-like structures. Compared to the localization profiles of CHMP2B with the T104N mutation in organelles, CHMP2B with mutations D148Y and Q165X (associated with the predominantly FTD phenotype) seemed unlikely to be localized in the Golgi body or in the ER and lysosome (see Figure S1), suggesting specific localization of CHMP2B with the T104N mutation (associated with the predominantly ALS phenotype) in the Golgi body.

### 3.2. CHMP2B with the T104N Mutation Inhibits Neuronal Morphological Differentiation

**Figure 3:**
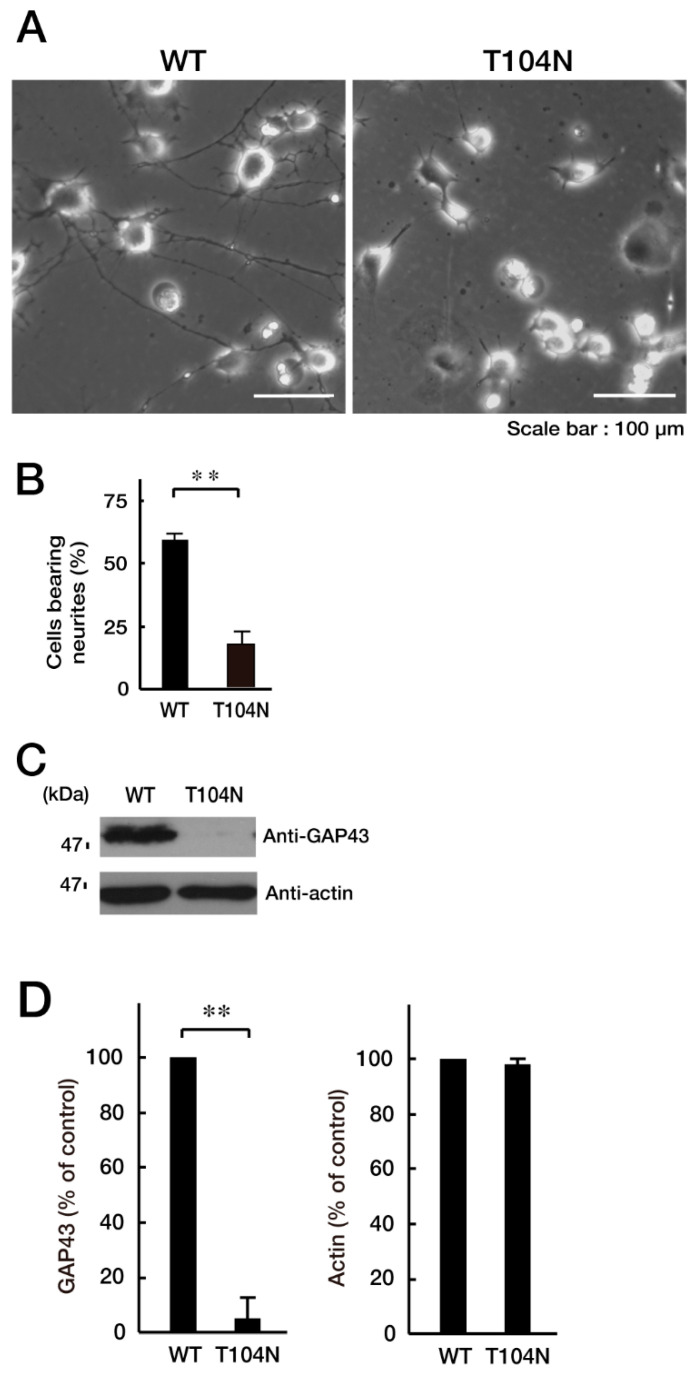
CHMP2B with the T104N mutation inhibits neuron-like process extension. N1E-115 cells with WT or mutated CHMP2B (T104N) were allowed to differentiate for 48 h. Typical cell images after 48 h are shown in (**A**). (**B**) Cells with processes with a body length greater than one cell were counted as cells with neurites and statistically shown in the graph (** *p* < 0.01; *n* = 10 fields). (**C,D**) The lysates of cells following the induction of differentiation (48 h) were immunoblotted with an antibody against neuron-specific marker GAP43 and actin as the internal marker protein, and their immunoreactive band intensities are statistically depicted (**D**) (** *p* < 0.01; *n* = 3 blots). WT—wild-type.

We explored whether CHMP2B with the T104N mutation affected neuronal morphological changes in N1E-115 cells, a differentiation model often used to study process elongation [15,16]. Briefly, cells with processes longer than the cell body length were counted as differentiated cells. Cells with WT CHMP2B demonstrated process elongation. In contrast, cells harboring CHMP2B with the T104N mutation failed to undergo sufficient process elongation (Figure 3A,B) and showed decreased expression of the neuronal marker growth-associated protein 43 (GAP43) (Figure 3C,D).

### 3.3. Attenuating Golgi Stress Recovers an Inhibitory Morphological Differentiation Phenotype

**Figure 4:**
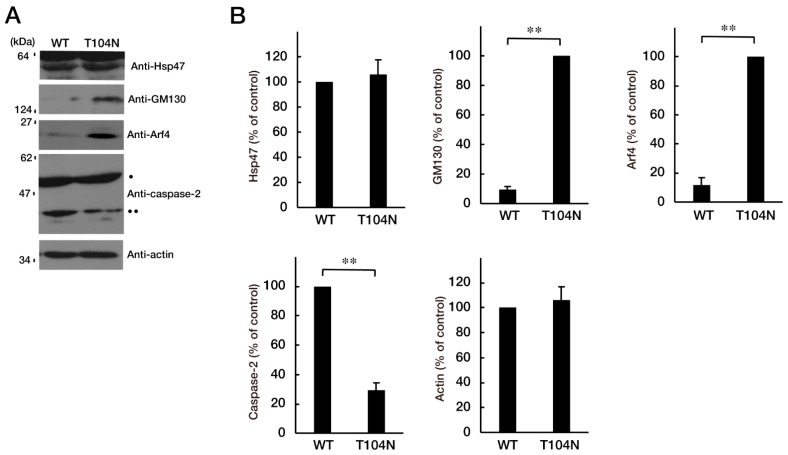
CHMP2B with the T104N mutation upregulates Golgi stress signal. (**A**,**B**) N1E-115 cells with WT or mutated CHMP2B (T104N) were allowed to differentiate for 48 h and lysed. The lysates were immunoblotted with an antibody against Golgi stress marker Hsp47, GM130, Arf4, cleaved caspase-2 (indicated with ••; pro-caspase-2 indicated with •), or control actin, and their immunoreactive band intensities are statistically depicted in the graph (** *p* < 0.01; *n* = 3 blots). WT—wild-type.

**Figure 5:**
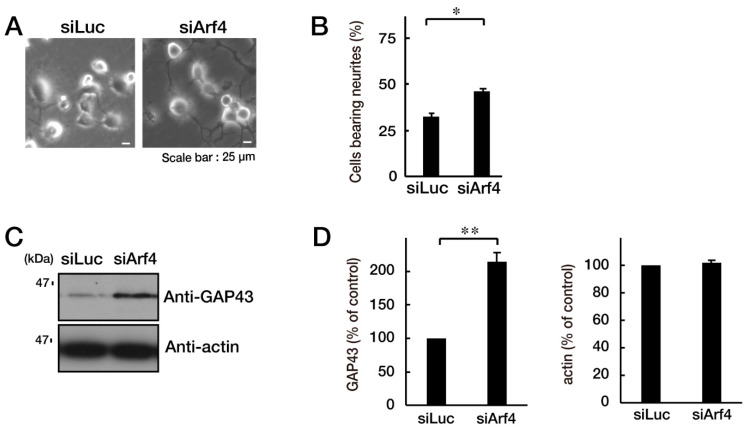
Knockdown of Arf4 recovers the phenotypes of cells with CHMP2B with the T104N mutation. N1E-115 cells with mutated CHMP2B were transfected with control luciferase (Luc) or Arf4 siRNA and allowed to differentiate for 48 h. Typical cell images after 48 h are shown in (**A**). (**B**) Cells with processes of more than one cell body length were counted as cells with neurites and are statistically depicted in the graph (* *p* < 0.05; *n* = 10 fields). (**C**,**D**) The lysates were immunoblotted with an antibody against GAP43 or control actin, and their immunoreactive band intensities are statistically depicted in the graph (** *p* < 0.01; *n* = 3 blots).

Because the mutated CHMP2B protein, but not the WT protein, was localized in the Golgi body, we wanted to determine which pathway related to Golgi stress was responsible for the expression of the mutated protein. Golgi stress is composed of pathways mediated by heat shock protein 47 (HSP47); transcription factor E3 (TFE3) and possible downstream targets such as structural proteins composing the Golgi body including GM130; cAMP response element binding protein (CREB) 3 and downstream Arf4; and caspase-2 [17,18,19,20,21,22,23,24]. Expression of mutated proteins led to increased levels of GM130 and Arf4 (Figure 4A,B), indicating that these pathways may be related to the Golgi stress response. In contrast, the amounts of cleaved (active) caspase-2 were decreased following expression of the mutated protein. It is possible that GM130 is a structural protein of the Golgi body and that its decreased expression could be associated with sustaining Golgi organelle structure. Because Arf4 is classified as a signal transducer molecule, its knockdown might affect the Golgi stress response, more directly. As expected, knockdown of Arf4 (Figure S2) recovered the mutant-induced inhibition of process elongation (Figure 5A,B). GAP43 expression levels were similarly recovered (Figure 5C,D). Thus, mutated CHMP2B proteins trigger Golgi stress, at least in part through Arf4, and knockdown of Arf4 can ameliorate the mutant-induced inhibition of morphological differentiation.

## 4. Discussion

**Figure 6:**
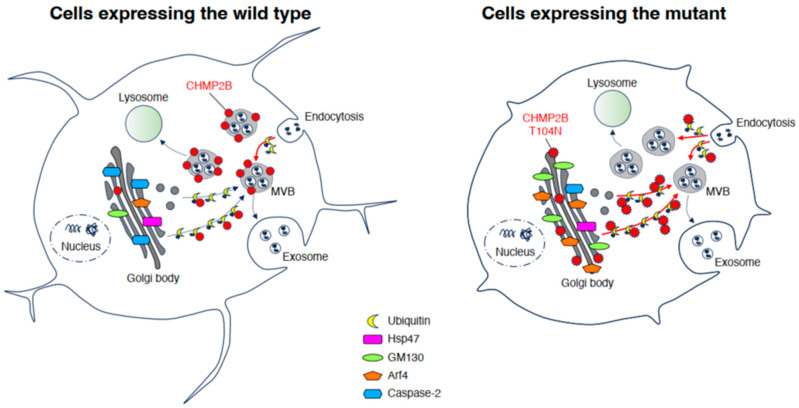
Schematic diagram of molecules associated with Golgi stress in wild-type and mutated CHMP2B-expressing cells. WT CHMP2B is localized with MVB, whereas mutated CHMP2B is preferentially colocalized with the Golgi body and increases stress signaling through Arf4. MVB, multivesicular body; Hsp47, heat shock protein 47.

This study demonstrated that the WT CHMP2B protein was colocalized with MVB-like structures, whereas the T104N mutated protein formed aggregates with ubiquitin protein. In addition, we provide the first evidence that the T104N-mutated protein colocalized with the Golgi body and inhibited the elongation of neurons. The mutated CHMP2B protein is related to Golgi stress via GM130 and Arf4 upregulation and caspase-2 downregulation, as Golgi stress markers (Figure 6).

FTD and ALS are neurodegenerative diseases with overlapping symptoms and causes; as such, they are commonly considered a single-spectrum disorder. Abnormalities in the homeostasis of biomaterials involving dysfunctional protein clearance, impaired RNA metabolism, and aberrant formation of complexes of proteins with RNA are emerging as key events underlying FTD/ALS pathogenesis. It is likely that these processes, including protein and nucleotide clearance, interact with each other at the molecular level, converging on a common molecular pathogenic pathway [25,26,27,28]. Studies on FTD/ALS7-associated CHMP2B reveal that CHMP2B preferentially participates in protein clearance [11,12]. Recent findings show that CHMP2B regulates casein kinase 1 (CK1) phosphorylation of the TAR DNA-binding protein of 43 kDa (TDP-43), which binds to RNAs as well as some DNAs [29]. TDP-43 is also involved in FTD/ALS [30]. Detailed analyses of the pathogenic mutations in CHMP2B will allow us to identify the relationship between CHMP2B and RNA metabolism through TDP-43. In the present study, WT CHMP2B typically displayed MVB-like structures in cells; in contrast, the T104N mutant protein exhibited aggregate-like structures; however, it is unknown whether these structures exist in the Golgi body as an aggregate-like complex containing RNA or protein (Figure 1A,B and Figure 2). Also, we demonstrated that ubiquitin was colocalized in WT and T104N protein (Figure 1C). Ubiquitin is known as an aggregate protein marker. The T104N CHMP2B protein was colocalized with ubiquitin, suggesting that proteins taken up by endocytosis were ubiquitinated by ESCRT3, which recognized the aggregated ubiquitinated proteins. WT CHMP2B also colocalized with ubiquitin, suggesting that it recognized ubiquitinated proteins taken up by endocytosis or Golgi vesicles. To clarify what is included in MVBs, further study, including exosome subtype analysis, is warranted. Additionally, it is unclear whether the structure directly exhibits toxic gain-of-function in cells. If protein aggregates gain cellular toxicity, apoptosis or Golgi stress may occur within the cell.

It is more likely that a common pathological molecular mechanism of the FTD/ALS spectrum is impaired proteostasis, especially for abnormal trafficking coupling endosome to lysosome. Among the protein components that constitute these proteostasis pathways, CHMP2B is a core component of the ESCRT machinery and coordinates the scission of intracellular membranes such as endosome membranes. The ESCRT is composed of sequential subcomplexes, ESCRT-0 to ESCRT-III, and facilitates endosome to in almost all cell types, including neuronal cells [9,10]. Before and during the formation of endosomes involving MVB, ESCRT plays a key role in intracellular membrane transport and remodeling, primarily at endosomes [9,10]. The sequential binding and function of ESCRT to the ubiquitinated proteins sequester them within the internal vesicles of the MVB. The ESCRT-III subcomplex involving CHMP2B shapes their membranes, cooperates with vacuolar protein sorting-associated protein (VPS) 4 as the ATPase, and undergoes fission of the membrane neck from inside the endosome. The ESCRT-0 to -II subcomplexes mainly contribute to the formation of the ESCRT-III subcomplex [9,10]. Therefore, mutations in CHMP2B of the ESCRT-III subcomplex give rise to several neurodegenerative diseases because all ESCRT protein components are required for appropriate morphogenesis and function of neuronal cells [9,10,11,12]. Because the CHMP2B protein with the T104N mutation exhibits aggregate-like structures but not MVB-like structures, this mutant protein seems unlikely to be functional within the ESCRT system. Exosomes are secreted via MVBs, whereas microvesicles form from the endoplasmic reticulum that buds directly from the plasma membrane. At this stage, no method has been established to reliably distinguish exosomes from microvesicles, and it is difficult to determine whether differences exist in exosome production between WT and T104N-mutated CHMP2B. If this hypothesis is true, it is conceivable that the loss of function of CHMP2B underlies the cellular basis of FTD/ALS pathogenesis.

The Golgi stress pathway is thought to mitigate the effects of specific stresses within cells or to arrest the cell cycle, as seen in the unfolded protein response (UPR) established in the ER. Increasing evidence indicates that Golgi stress is mediated by four major pathways: (1) HSP47, (2) TFE3 and possible downstream targets such as the structural proteins composing the Golgi body, (3) CREB3 and Arf4, and (4) caspase-2 [17,18,19,20,21,22,23,24]. First, HSP47 is an ER chaperone, and it is likely that HSP47 is also localized in the Golgi body. HSP47 is thought to protect the Golgi body from various stresses [17,18,19,20,21,22,23,24], and expression levels of HSP47 were comparable in cells expressing WT and mutant CHMP2B. Second, TFE3 is an essential transcription factor controlling the genes that encode Golgi body structural proteins such as GM130, intracellular vesicle transporting molecules, and Golgi-resident enzymes mediating glycosylation [17,18,19,20,21,22,23,24]. Because GM130 is upregulated in cells expressing mutated CHMP2B, it is thought that its pathway through TFE3 is responsible for the Golgi stress induced by mutated CHMP2B proteins. Third, the specific targeting pathway of the transcription factor CREB3 is the small GTPase Arf4 [17,18,19,20,21,22,23,24]. Arf4 is upregulated in cells expressing mutated CHMP2B. The pathway through Arf4 may be responsible for Golgi stress induced by mutated CHMP2B proteins. Fourth, active caspase-2 mainly plays a non-apoptotic role, lacking the ability to activate effector caspases such as caspase-3. Procaspase-2 is also present on the cytoplasmic surface of the Golgi body. The prodomain of procaspase-2 is cleaved to generate active caspase-2, probably suppressing the functions of the Golgi body by cleaving proteins such as golgin-160 [17,18,19,20,21,22,23,24]; however, cleaved active caspase-2 results in a decrease in cells expressing mutated CHMP2B, indicating that the pathway through caspase-2 is not involved in Golgi stress in cells expressing mutated CHMP2B. Caspase-2 has been linked to lipid metabolism; however, lipid metabolism may also be involved in the T104N mutation (Figure 4). Further study is required to determine the expression of 3-hydroxy-3-methylglutaryl coenzyme A (HMG-CoA), low-density lipoprotein receptor (LDLr), FAS, and farnesyl diphosphate synthase (FPP synthase) [31]. We focused only on the pathway through CREB3 and Arf4, because the pathway through TFE3 and molecules such as GM130 significantly contributes to the homeostasis of the Golgi body [32]. As expected, knockdown of Arf4, possibly acting through decreasing Golgi stress, recovered morphological differentiation with process elongation in cells expressing mutated CHMP2B.

Arf4 is a small GTPase thought to participate in intracellular vesicle trafficking with other Arf-family small GTPases. The Arf family of small GTPases is composed of classes I (Arf1 and Arf2 and/or Arf3), II (Arf4 and Arf5), and III (Arf6). Class I GTPases such as Arf1 regulate the vesicle transport system around the Golgi body, and the class III GTPase Atf6 primarily controls vesicle transport around the intracellular surface of the plasma membrane. In contrast, the precise role of the class II GTPase Arf4 remains unclear [33,34]. For example, Ezratty et al. reported that Arf4 regulates polarized exocytosis by acting through the complex between the basal body and the ciliary body. It is thus likely that Arf4 regulates Notch signaling in epidermal morphological differentiation [35]. Wang et al. reported that Arf4 and the guanine-nucleotide exchange factor GBF1 synergize with the sensory receptor cargo to regulate trafficking of the ciliary membrane [36]. It is thought that the activities of Arf4 are precisely associated with the formation of the ciliary membrane [37,38]. It will be important to determine which organelles in the cognate intracellular membrane transport are mediated by Arf4 and how this is achieved. In addition, it remains unclear how signaling around Arf4 is related to the response to Golgi stress. 

The question of why and how CHMP2B is involved in promoting process elongation remains to be answered (Figure 5). In the initial steps of neuronal morphological differentiation, cells undergo dynamic morphogenesis such as neurite outgrowth and elongation. Dynamic morphogenesis requires the synthesis of many membrane lipids and proteins to achieve neurite outgrowth and elongation. Therefore, during cell development, a quality control step of protein is required; that is, proteostasis. It is possible that CHMP2B, as the ESCRT-III subcomplex component, and other ESCRT components directly or indirectly monitor fine-tuned neurite outgrowth and elongation. Here we show that the FTD/ALS7-associated mutation of CHMP2B inhibits neuronal morphological differentiation. Mutated CHMP2B is specifically localized in the Golgi body to trigger Golgi stress. In contrast, knockdown of Arf4, a Golgi stress mediator, recovers the ability of cells to differentiate. Further studies on the relationship of mutated CHMP2B with Golgi stress are needed to increase our understanding of the detailed mechanisms by which the FTD/ALS7-associated mutation of CHMP2B inhibits neuronal morphological differentiation using cells and genetically modified mice, as well as of a possible causal relationship between inhibitory differentiation and the early stages of neurodegeneration in FTD/ALS7. Additional studies will allow us to elucidate the role of Golgi stress as a potential molecular and cellular pathological mechanism underlying FTD/ALS7 and might lead to the development of therapeutic target–specific drug candidates for FTD/ALS7 and other types of FTD/ALS.

This study investigated the role of CHMP2B in neuronal cell biology. There are several types of FTD/ALS, and cellular disorders related to the causative gene have been reported [39,40]. Further investigation of the changes caused by the causative genes in other diseases may lead to the establishment of new therapeutic targets for FTD/ALS if common phenomena can be found among the different types of FTD/ALS.

## Figures and Tables

**Table 1:** Key antibodies and plasmids used in this study.

Reagent or Material	Company or Source	Cat. No.	Lot. No.	Concentration Used
Antibody				
Anti-heat shock protein (HSP) 47	Santa Cruz Biotechnology	sc-5293	I2118	Immunoblotting (IB), 1/200
Anti-Arf4	Proteintech	11673-1-AP	00048284	IB, 1/1000
Anti-caspase-2	Abcam	ab179520	GR209449-2	IB, 1/1000
Anti-actin	MBL	M177-3	007	IB, 1/5000
Ant-growth-associated protein 43 (GAP43)	Santa Cruz Biotechnology	sc-17790	J0920	IB, 1/5000
Anti-Lys-Asp-Glu-Leu (KDEL)	MBL	M181-3	004	Immunofluorescence (IF), 1/200
Anti-130 kDa Golgi membrane protein (GM130)	BD Biosciences	610823	8352796	IB, 1/500 and IF, 1/200
Anti-cathepsin D	Abcam	ab75852	GR260148-33	IF, 1/200
Anti-IgG (H+L chain) (Rabbit) pAb-HRP	MBL	458	353	IB, 1/5000
Anti-IgG (H+L chain) (Mouse) pAb-HRP	MBL	330	365	IB, 1/5000
Goat pAb to Ms IgG (Alexa Fluor 488 conjugate)	abcam	ab150113	GR173498-1	IF, 1/500
Alexa Fluor TM 594 goat anti-mouse IgG (H+L)	invitrogen	A11005	226-8383	IF, 1/500
Alexa Fluor TM 488 goat anti-rabbit IgG (H+L)	invitrogen	A11008	075-1094	IF, 1/500
Alexa Fluor TM 594 goat anti-rabbit IgG (H+L)	invitrogen	A11012	201-8240	IF, 1/500
Recombinant DNA				
pEGFP-C1-human CHMP2B	Generated in this study	Not applicable		1.25 μg of DNA per 6 cm dish
pEGFP-C1-human CHMP2B with the T104N mutation	Generated in this study	Not applicable		1.25 μg of DNA per 6 cm dish

## Data Availability

Not applicable.

## Supplementary Materials

The following supporting information can be downloaded at: https://www.mdpi.com/article/10.3390/neurolint15030063/s1, Figure S1: CHMP2B with the D148Y or Q165X mutation (showing predominant FTD phenotypes) displays aggregation-like structures; Figure S2: Comparison of knockdown efficiencies of siRNAs for Arf4; Figure S3: Original size images of Figure 3; Figure S4: Original size images of Figure 4; Figure S5: Original size images of Figure 5; Figure S6: Original size images of Figure S2.
