# Supplementary material for: FTD/ALS Type 7-Associated Thr104Asn Mutation of CHMP2B Blunts Neuronal Process Elongation, and Is Recovered by Knockdown of Arf4, the Golgi Stress Regulator
Source: Neurol Int. 2023 Aug 11;15(3):980–93. doi: 10.3390/neurolint15030063 (PMC10443297; doi:10.3390/neurolint15030063)
Supplement: Supplementary file 1 [file neurolint-15-00063-s001.zip › neurolint-2481820-supplementary.pdf]

Supplemental figure legends

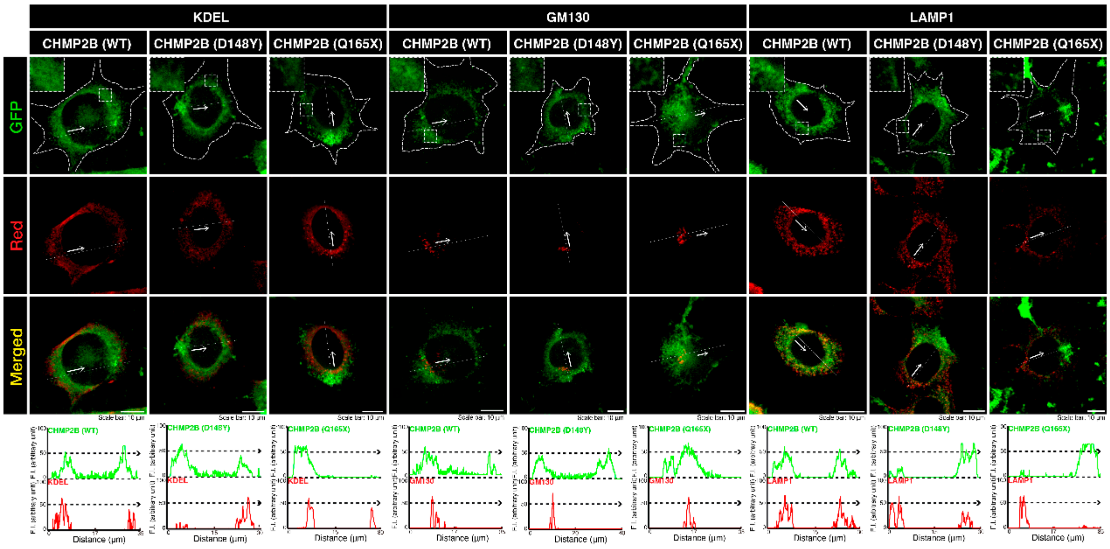

Figure S1. CHMP2B with the D148Y or Q165X mutation (showing predominant FTD phenotypes) displays aggregation-like structures. Cells were transfected with a plasmid encoding the GFP-tagged wild type (WT) or mutated CHMP2B (D148Y and Q165X). Transfected cells (green) were stained with an antibody against the ER-specific antigen KDEL (red), the Golgi body-specific antigen GM130 (red), or the lysosome- and the related organelles-specific LAMP1 (red). The approximate outlines of cells are shown by white dotted lines. Large magnified images surrounded with white squares are magnified versions of the small images surrounded with white squares. Scan plots were performed along the white lines in the direction of the arrows in the green and red images. Graphs showing the fluorescence intensities (F.I., arbitrary units) along the lines in the direction of the arrows are shown in the bottom panels. GFP, green fluorescent protein; WT, wild-type; ER, endoplasmic reticulum.

A

siRNA Sequences (5' to 3')

Sense chain for siLuciferase	GCCAUUCUAUCCUCUAGAG-dTdT
Antisense chain for siLuciferase	CUCUAGAGGAUAGAAUGGC-dTdT
Sense chain for siArf4-90th	GACGACAAUUCUGUAUAAA-dTdT
Antisense chain for siArf4-90th	UUUAUACAGAAUUGUCGUC-dTdT
Sense chain for siArf4-214th	GAUAAAAUUAGGCCUCUCU-dTdT
Antisense chain for siArf4-214th	AGAGAGGCCUAAUUUUAUC-dTdT
Sense chain for siArf4-296th	GAAUCCAGGAAGGAGCAGC-dTdT
Antisense chain for siArf4-296th	GCUGCUCCUUCCUGGAUUC-dTdT
Sense chain for siArf4-340th	GAUGAGCUGCAGGAUGCAG-dTdT
Antisense chain for siArf4-340th	CUGCAUCCUGCAGCUCAUC-dTdT

B

DNA Primers (5' to 3')

Sense primer for actin	ATGGATGACGATATCGCTGCGCTGGTC
Antisense primer for actin	CTAGAAGCACTTGCGGTGCACGATGGAG
Sense primer for Arf4	ATGGGCCTCACCATCTCCTC
Antisense primer for Arf4	TTAACGTTTTGAAAGTTCATTTGACAGCC

C

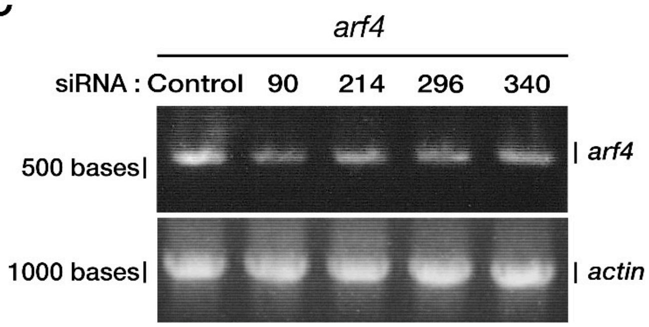

Figure S2. Comparison of knockdown efficiencies of siRNAs for Arf4. (A-C) Cells were transfected with the respective siRNAs (the numbers indicate nucleotides from A^1TG^3) in accordance with the manufacturer's instructions. Total RNAs were used for RT-PCR with the indicated primers to evaluate their respective knockdown efficiencies. The 90th Arf4 siRNA was used for experiments. RT-PCR for the control gene, actin, is also shown. siRNA, small interfering RNA; RT-PCR, reverse transcription polymerase chain reaction.

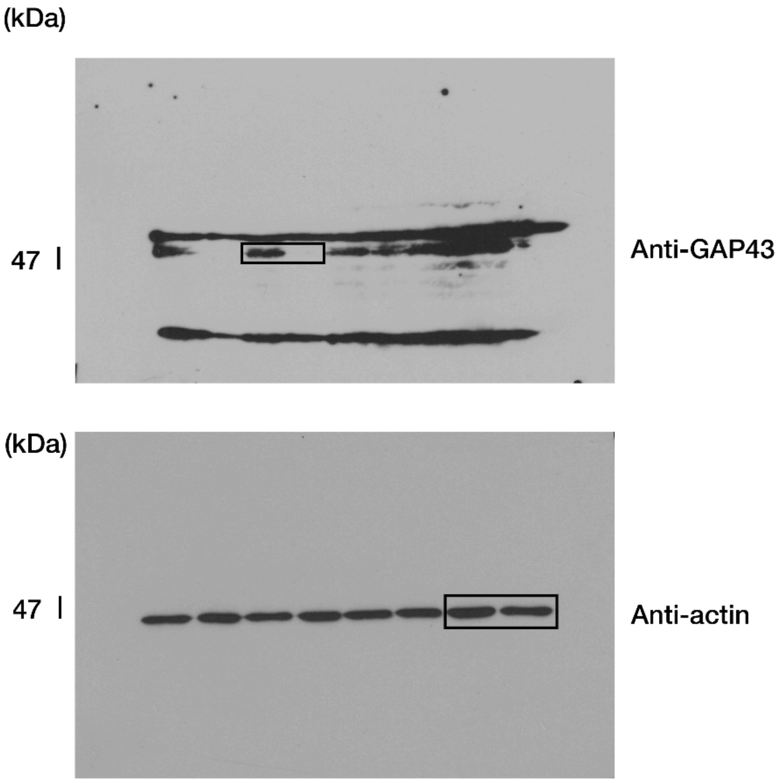

Figure S3. Original size images of Figure 3.

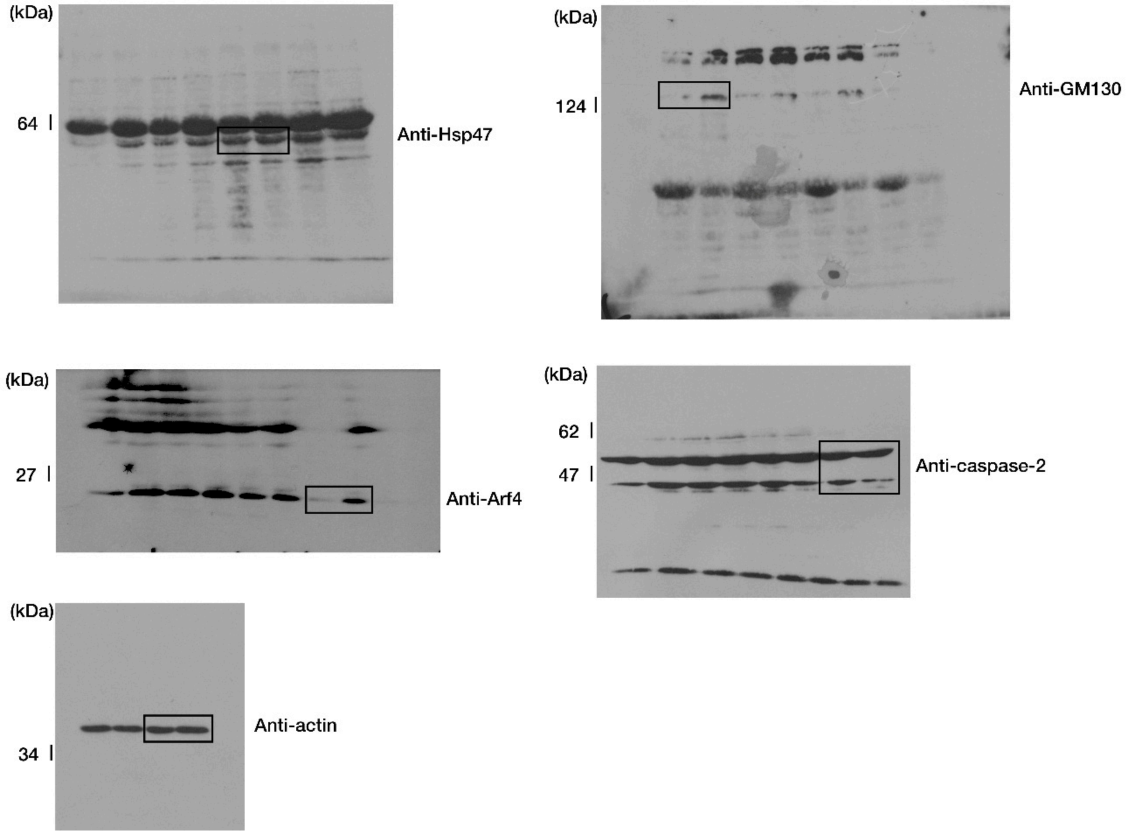

Figure S4. Original size images of Figure 4.

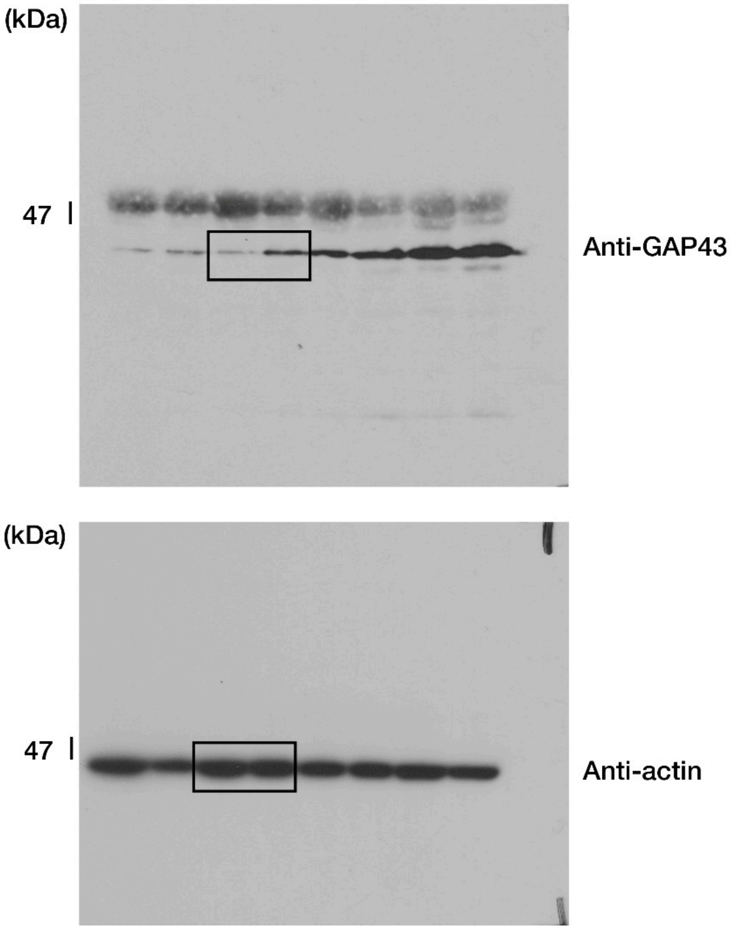

Figure S5. Original size images of Figure 5.

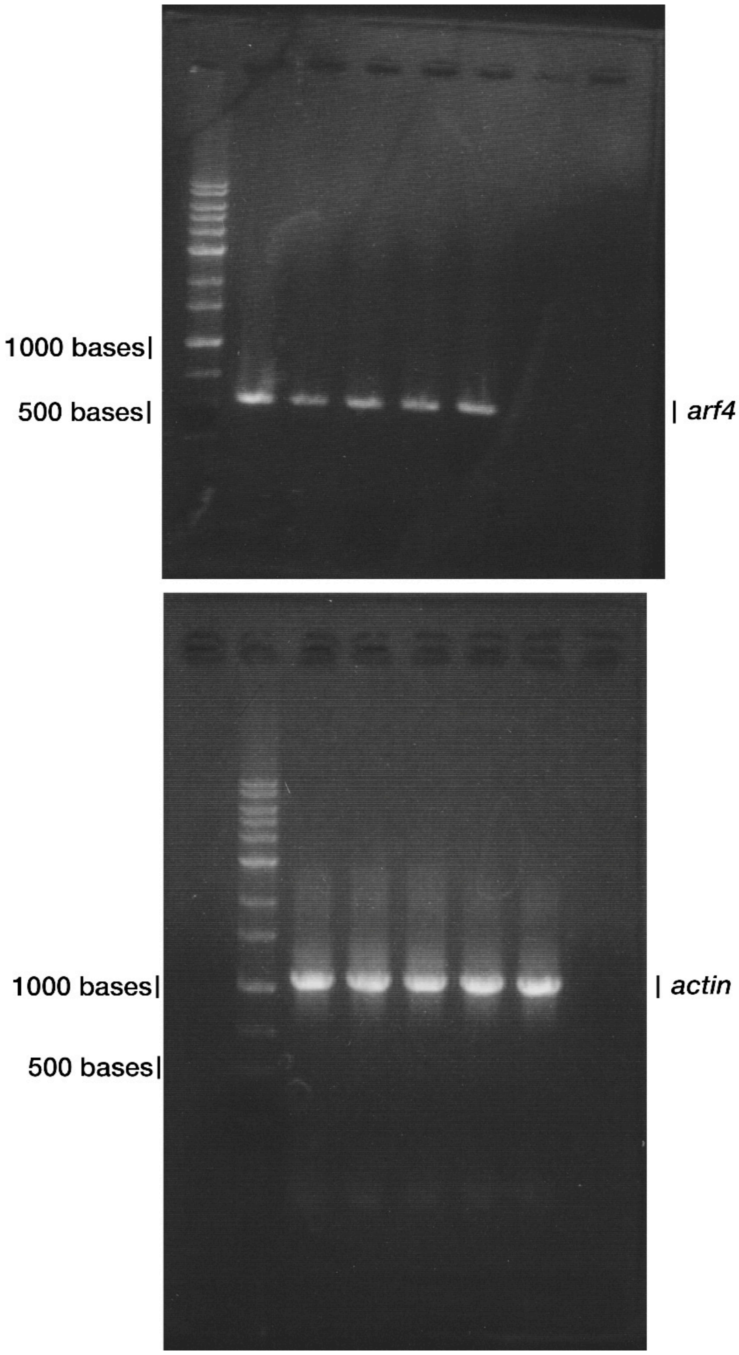

Figure S6. Original size images of Figure S2.
